# Distinctive Deep‐Level Defects in Non‐Stoichiometric Sb_2_Se_3_ Photovoltaic Materials

**DOI:** 10.1002/advs.202105268

**Published:** 2022-01-25

**Authors:** Weitao Lian, Rui Cao, Gang Li, Huiling Cai, Zhiyuan Cai, Rongfeng Tang, Changfei Zhu, Shangfeng Yang, Tao Chen

**Affiliations:** ^1^ Hefei National Laboratory for Physical Sciences at Microscale CAS Key Laboratory of Materials for Energy Conversion Department of Materials Science and Engineering School of Chemistry and Materials Science University of Science and Technology of China Hefei Anhui 230026 China; ^2^ Institute of Energy Hefei Comprehensive National Science Center Hefei China

**Keywords:** antimony triselenide, carrier lifetime, deep‐level defect, DLTS, SRH recombination

## Abstract

Characterizing defect levels and identifying the compositional elements in semiconducting materials are important research subject for understanding the mechanism of photogenerated carrier recombination and reducing energy loss during solar energy conversion. Here it shows that deep‐level defect in antimony triselenide (Sb_2_Se_3_) is sensitively dependent on the stoichiometry. For the first time it experimentally observes the formation of amphoteric Sb_Se_ defect in Sb‐rich Sb_2_Se_3_. This amphoteric defect possesses equivalent capability of trapping electron and hole, which plays critical role in charge recombination and device performance. In comparative investigation, it also uncovers the reason why Se‐rich Sb_2_Se_3_ is able to deliver high device performance from the defect formation perspective. This study demonstrates the crucial defect types in Sb_2_Se_3_ and provides a guidance toward the fabrication of efficient Sb_2_Se_3_ photovoltaic device and relevant optoelectronic devices.

## Graphical abstract

Amphoteric Sb_Se_ defect in theoretical prediction, possessing equivalent capability of trapping electron and hole, plays a critical role in charge recombination and device performance. It experimentally discloses the existence of amphoteric Sb_Se_ defect for the first time, and establishes the correlation between defect properties, charge recombination, carrier dynamics as well as device performance using deep level transient spectroscopy.

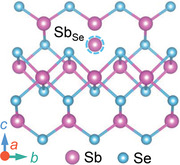

## 1 Introduction

In semiconducting materials, point defect with deep energy level and large capture cross section possesses high probability to induce trap‐assisted Shockley‐Read‐Hall (SRH) recombination.^[^ ^1^, ^2^, ^3^ ^]^ It is also recognized that SRH recombination degrades the solar cell performance by inducing open‐circuit voltage (*V*_OC_) loss,^[^ ^4^ ^]^which is the major challenge in the materials synthesis for high performance solar cells. Therefore, the identification and passivation of deep‐level defects arouse intense interests. The continuous efforts in the field, from Cu(In,Ga)Se_2_ and CdTe to perovskite solar cells, have upgraded the solar cell fabrication technologies and new understandings in the semiconducting materials.^[^ ^4^, ^5^, ^6^, ^7^ ^]^

As a class of emerging photovoltaic material, quasi‐1D antimony triselenide (Sb_2_Se_3_) has drawn increasing interests due to its suitable band gap (1.1–1.3 eV), large light absorption coefficient (>10^5^ cm^−1^ in visible and near‐infrared region), earth‐abundant storage, low toxicity and benign grain boundaries without dangling bonds along (Sb_4_Se_6_)_n_ ribbons.^[^ ^8^, ^9^, ^10^ ^]^ Recent years, power conversion efficiency (PCE) of Sb_2_Se_3_ has made a great progress, Li et al. achieved the highest PCE of 9.2%,^[^ ^11^ ^]^ indicating the great potential for future application upon further efficiency improvement. In some well‐prepared devices, the short‐circuit current density (*J*_SC_) and fill factor (*FF*) can reach decent values. However, all reported *V*_OC_ of Sb_2_Se_3_ solar cells is below 500 mV, corresponding to a serious *V*_OC_ loss of 700–800 mV.^[^ ^8^, ^12^, ^13^ ^]^ Therefore, the large *V*_OC_ deficit has become the major limiting factor for essential efficiency improvement. In principle, to improve the *V*_OC_, it is critical to suppress the SRH recombination induced by deep‐level defect.^[^ ^4^, ^13^ ^]^

The theoretical studies have pointed out that there are complicated deep‐level defects derived from the distinctive quasi‐1D structure in Sb_2_Se_3._^[^ ^13^, ^14^, ^15^, ^16^, ^17^, ^18^ ^]^ Practically, it is difficult to serve as guidance for the solar cell fabrication since the calculations consider all possible defects regardless of the materials processing. The materials synthesis usually influences the defect formation. Therefore, it is an urgent task to probe the defect characteristics experimentally. In this regard, there are three issues need to be resolved: (1) high‐purity sample should be prepared for clear and reliable detection, (2) the defect should be correlated with device performance, which requires specific detection technique, and (3) the relationship between defect and carrier dynamics is required to be established to sophistically control carrier transport and suppress recombination.

Here we figure out the critical issues by fabricating high purity Sb‐rich and Se‐rich Sb_2_Se_3_ films, in which only the Sb, Se and Sb_2_Se_3_ are applied as the precursor materials, so as to gain films without impurities derived from extrinsic elements. The deep‐level transient spectroscopy (DLTS) measurement is based on complete device, which perfectly establish the defect properties and device performance. We further investigate the correlations between the defect properties and carrier lifetime which is characterized by transient absorption spectroscopy (TAS). Therefore, we are able to finally establish the correlation between materials processing, defect properties, carrier recombination dynamics as well as the device performance, and in turn provide new understanding to manufacture Sb_2_Se_3_ for efficient device applications.

## 2 Results and Discussion

**Figure 1:**
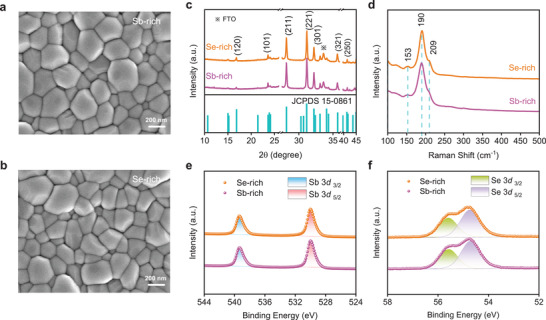
a,b) Surface SEM images of Sb‐rich and Se‐rich Sb_2_Se_3_ films. c,d) XRD patterns and Raman spectra of Sb‐rich and Se‐rich Sb_2_Se_3_ films. e,f) XPS spectra of Sb 3*d* and Se 3*d* for Sb‐rich and Se‐rich Sb_2_Se_3_.

In brief, we prepare Sb‐rich and Se‐rich Sb_2_Se_3_ films by using Sb or Se with Sb_2_Se_3_ powders as precursor materials in a dual‐source thermal evaporation deposition (see details in Experimental Section). Finally, the atomic ratio of Se/Sb for Sb‐rich and Se‐rich Sb_2_Se_3_ are 1.37 and 1.56 measured by energy dispersive X‐ray spectroscopy (EDX), respectively (Table S1, Supporting Information). SEM images (**Figure** **1**a,b, Figure S1a,b, Supporting Information) indicate that both Sb‐rich and Se‐rich films show compact and pin‐hole free morphology. Furthermore, the atomic force microscopy (AFM) images also display the similar roughness (root mean square, *R*_q_) for Sb‐rich and Se‐rich Sb_2_Se_3_ films (Figure S2, Supporting Information), which are 25.4 and 25.9 nm, respectively. Notably, all diffractions of XRD patterns (Figure 1c) are consistent well with [hk1] preferentially oriented orthorhombic Sb_2_Se_3_ (JCPDS No. 15‐0861),^[^ ^11^ ^]^ and no noticeable diffraction peaks of impurities such as elemental Sb, Se and oxides are observed.

To further confirm the purity of the samples, we conduct the Raman spectroscopy and X‐ray photoelectron energy spectroscopy (XPS). Raman spectra (Figure 1d) demonstrate that both Sb‐rich and Se‐rich films only display three characteristic peaks at 153 (*B*_g_), 190 (*A*_g_), and 209 (*A*_g_) cm^−1^, which are indexed to the symmetric vibration of Sb—Se bond in Sb_2_Se_3_ respectively.^[^ ^19^ ^]^ Obviously, there are no peaks belonging to Sb_2_O_3_ detected.^[^ ^20^ ^]^ In addition, the XPS spectra (Figure 1e,f) appear two peaks at high binding energy of 539.3 and 529.9 eV, which are ascribed to the Sb 3*d*_3/2_ and 3*d*_5/2_ of Sb_2_Se_3_. The low binding energy at 55.6 and 54.7 eV are corresponding to Se 3*d*_3/2_ and 3*d*_5/2_ of Sb_2_Se_3_,^[^ ^20^ ^]^ respectively. Combining the XRD and XPS results, we confirm that the as‐prepared Sb_2_Se_3_ films are free of impurities such as elemental Sb, elemental Se and oxides.^[^ ^21^ ^]^

Furthermore, to determine the band gap and energy levels of the as‐synthesized Sb_2_Se_3_ films, we conduct ultraviolet–visible (UV–vis) absorption spectroscopy and ultraviolet photoelectron energy spectroscopy (UPS). UV–vis spectra (Figure S1d, Supporting Information) demonstrate that Sb‐rich and Se‐rich Sb_2_Se_3_ share an identical band gap of 1.20 eV. Then we measured secondary electron cutoff and valence band position through UPS spectra (Figure S1e,f, Supporting Information). Therefore, we can calculate the work function (Fermi level) of Sb‐rich and Se‐rich Sb_2_Se_3_ which are −4.64 and −4.67 eV, respectively. Combined with the band gap of 1.20 eV, we can calculate that the conduction band minimum (CBM) and valence band maximum (VBM) position of Sb‐rich Sb_2_Se_3_ are −4.25 and −5.45 eV, while the CBM and VBM of Se‐rich Sb_2_Se_3_ are −4.26 and −5.46 eV, respectively.

To further identify the conductivity type of Sb_2_Se_3_, we also carry out Hall effect measurement. The as‐prepared Sb_2_Se_3_ films with 300‐nm thickness and 1‐cm^2^ area are selected as samples to be examined at 300 K. And the results are summarized in Table S2 (Supporting Information). The Sb‐rich and Se‐rich Sb_2_Se_3_ show similar carrier concentration (≈10^16^ cm^−3^) and both possess negative Hall coefficient. The negative Hall coefficient indicates the majority carriers of Sb_2_Se_3_ are electrons, namely, N type conductivity.

**Figure 2:**
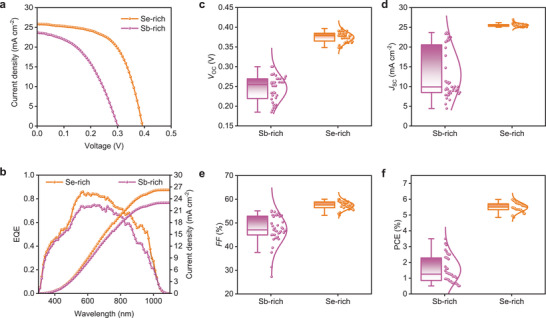
a) *J–V* curves of the champion Sb‐rich and Se‐rich Sb_2_Se_3_ solar cells. b) EQE characteristics and the corresponding integrated current density for optimal Sb‐rich and Se‐rich Sb_2_Se_3_ devices. c–f) Statistical *V*_OC_, *J*_SC_, *FF*, and PCE of Sb‐rich and Se‐rich devices.

The DLTS characterization is based on complete device structure, it is thus necessary to examine the photovoltaic performance of the films to confirm the quality at device level. The solar cell with a superstrate configuration of FTO/CdS/Sb_2_Se_3_/Spiro‐OMeTAD/Au was fabricated (Figure S1c, Supporting Information). The current density–voltage (*J–V*) characteristics for optimal Sb‐rich and Se‐rich devices were examined under standard AM1.5G illumination (**Figure** **2**a). The statistical *V*_OC_, *J*_SC_, *FF*, and PCE of 40 devices each for Sb‐rich and Se‐rich Sb_2_Se_3_ are illustrated in Figure 2c–f. Strikingly, Se‐rich Sb_2_Se_3_ device shows more efficient performance than Sb‐rich Sb_2_Se_3_ devices, especially the remarkable enhancement of *V*_OC_ and *FF*. The excellent performance reproducibility for Se‐rich devices suggests the reliability of the device fabrication. Finally, the best Se‐rich device delivers a PCE of 6.0%, with *V*_OC_ of 390 mV, *J*_SC_ of 25.86 mA cm^−2^ and *FF* of 59.21%. In contrast, the Sb‐rich device only delivers a maximum PCE of 3.5%, with *V*_OC_ of 300 mV, *J*_SC_ of 23.39 mA cm^−2^ and *FF* of 49.79%, respectively.

Moreover, we also perform electrochemical impedance spectroscopy (EIS) to study the resistance of Sb_2_Se_3_ device (Figure S3, Supporting Information). The charge transfer resistance (*R*_s_) and charge recombination resistance (*R*_rec_) for Sb‐rich and Se‐rich Sb_2_Se_3_ can be extracted from the arc of Nyquist plot according to corresponding equivalent circuit. The *R*_s_ for Sb‐rich and Se‐rich Sb_2_Se_3_ devices are 3.81 and 2.66 Ω cm^2^, while the *R*_rec_ for Sb‐rich and Se‐rich Sb_2_Se_3_ devices are 42.66 and 126.12 Ω cm^2^, respectively. In principle, the reduced *R*_s_ reflects the improved interface, and the enhanced *R*_rec_ indicates the inhibited trap‐assisted SRH recombination. Therefore, it suggests that Se‐rich device possesses better interface and lower bulk SRH recombination rate.

The external quantum efficiency (EQE, Figure 2b) is also carried out. The EQE results indicate that both kinds of devices show photoelectron response over a wide range from 300 to 1100 nm. In addition, the notch at 400–500 nm wavelength is associated with the parasitic absorption of CdS substrate.^[^ ^22^ ^]^ In particular, the increased response at long wavelength (500–1000 nm) for Se‐rich device is attributed to the improved electrical quality of Sb_2_Se_3_ film which will be analyzed from defect and carrier transport perspective later. Furthermore, the integrated *J*_SC_ of Sb‐rich (22.98 mA cm^−2^) and Se‐rich (26.26 mA cm^−2^) devices obtained from EQE accords well with that of *J*–*V* characteristics. Notably, the absorption onset for Sb‐rich and Se‐rich Sb_2_Se_3_ films calculated from differential EQE (Figure S4, Supporting Information) both are 1010 nm, which is corresponding to 1.23 eV band gap according to *E*_g_ = hc/*λ* (where *E*_g_, h,c, and *λ* are respective for band gap, Planck constant, light speed and wavelength), and the result echoes the UV–vis spectra.

**Figure 3:**
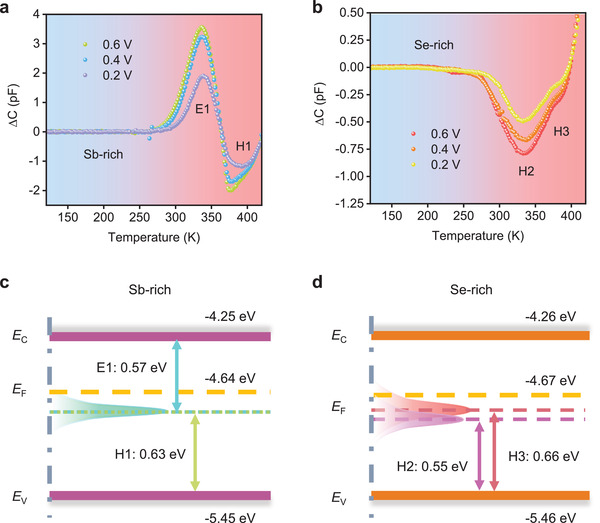
a,b) Dual‐pulse mode DLTS signals under variable pulse voltage (0.2–0.6 V) for Sb‐rich and Se‐rich Sb_2_Se_3_ devices. c,d) Schematic diagram of energy band and defect level of Sb‐rich and Se‐rich Sb_2_Se_3_. *E*_C_, *E*_V_, and *E*_F_ stand for CBM, VBM, and Fermi level, respectively.

We then perform DLTS characterizations, which are selected with optical and electrical dual‐pulse mode. Multiple pulse voltages are applied thus to realize repeatable and convincing results. In addition, as for N type Sb_2_Se_3_, the positive peaks and negative peaks represent electron and hole traps, corresponding to donor and acceptor defects respectively. Therefore, as shown in **Figure** **3**a,b, there are one electron trap (E1) and one hole trap (H1) observed in Sb‐rich Sb_2_Se_3_ sample, while there are two hole traps (H2 and H3) identified in Se‐rich Sb_2_Se_3_. The detailed defect information including trap level (*E*_T_), capture cross section (*σ*) and trap density (*N*_T_) extracted from DLTS signals are summarized in **Table** **1**. In detail, the trap E1 lies at 0.57 eV below CBM, and the trap H1 is at 0.63 eV above VBM in Sb‐rich Sb_2_Se_3_ (depicted in Figure 3c). Interestingly, considering the band gap of 1.2 eV, they exactly lie at the same position (mid‐gap) in forbidden band, even though one of them manifests donor characteristic, the other displays acceptor property. Furthermore, the trap E1 and H1 also show similar trap density and capture cross section. Interestingly, a theoretical study of Savory and Scanlon revealed Sb_Se_ antisite defect in Sb‐rich Sb_2_Se_3_ is amphoteric and possesses equivalent capability of capturing electron and hole simultaneously, resulting in similar trap energy level, capture cross section and trap density for electron and hole.^[^ ^16^ ^]^ Accordingly, we can reasonably assign the two trap signals as one amphoteric defect. Furthermore, Sb_Se_ defect shows much high concentration (at ≈10^14^ cm^−3^ level), owing to its quite low defect formation energy compared with formation enthalpy of Sb_2_Se_3_ phase. Therefore, this defect plays crucial role in carrier recombination in solar cells.

**Table 1:** Summarized defect information obtained from DLTS signals of Sb‐rich and Se‐rich Sb_2_Se_3_

Sb_2_Se_3_	Trap	*E*_T_ [eV]	*σ* [cm^2^]	*N*_T_ [cm^−3^]
Sb‐rich	E1	*E*_C_−0.57 ± 0.04	(0.04–3.89) × 10^−15^	(0.05–2.68) × 10^14^
	H1	*E*_V_+0.63 ± 0.07	(0.03–1.20) × 10^−15^	(0.11–2.10) × 10^14^
Se‐rich	H2	*E*_V_+0.55 ± 0.06	(0.01–3.57) × 10^−15^	(0.23–2.09) × 10^13^
	H3	*E*_V_+0.66 ± 0.07	(0.03–5.03) × 10^−15^	(0.07–2.51) × 10^13^

**Figure 4:**
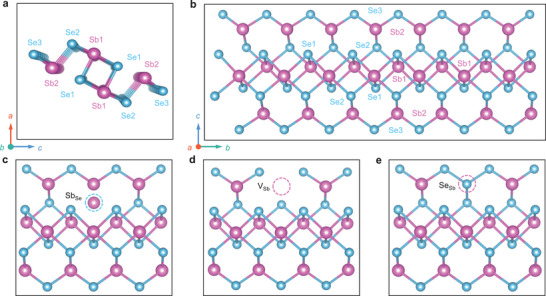
a,b) The perfect lattice of quasi‐1D Sb_2_Se_3_. c–e) The possible position of Sb_Se2_, V_Sb2_, and Se_Sb2_ defects in Sb_2_Se_3_ lattice respectively.

In Se‐rich Sb_2_Se_3_ sample, the traps H2 and H3 that locate at 0.55 and 0.66 eV above the VBM (Figure 3d) are respectively assigned to V_Sb_ vacancy and Se_Sb_ antisite defects according to the calculations,^[^ ^16^, ^17^, ^18^ ^]^ since their rather low formation energy and suitable transition level in Se‐rich Sb_2_Se_3_ sample. It should be noted that all the atomic positions Sb1, Sb2, Se1, Se2, and Se3 may generate the respective V_Sb_, Se_Sb_, and Sb_Se_ defects in each sample (**Figure** **4**a–e), since the same kind of defect at different atomic positions possess quite close formation energy and transition level in theoretical calculations.^[^ ^16^, ^17^, ^18^ ^]^

Evidently, no matter whether Sb_Se_ defect in Sb‐rich or V_Sb_ and Se_Sb_ defects in Se‐rich, all of them are ultra‐deep‐level defects with active energy much higher than 0.025 eV.^[^ ^16^, ^22^ ^]^ Therefore, they are hardly ionized but act as recombination centers, resulting in trap‐assisted SRH recombination, it is thus detrimental to carrier lifetime. In particular, the amphoteric Sb_Se_ defect in Sb‐rich Sb_2_Se_3_ possesses a similar ability of trapping electron and hole, which means the comparative possibility of pinning electron and hole quasi‐Fermi level, while the V_Sb_ and Se_Sb_ in Se‐rich are only associated with hole trapping. Furthermore, the higher defect density of Sb_Se_ compared with V_Sb_ and Se_Sb_ would generate more severe decline of carrier lifetime (*τ*) according to SRH model (*τ* ∝ (*σ N*_T_)^−1^).^[^ ^1^, ^2^ ^]^

**Figure 5:**
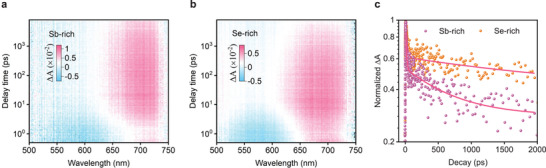
a,b) TAS mapping of Sb‐rich and Se‐rich Sb_2_Se_3_ films deposited on glass substrate. c) Transient decay kinetics (scatter) and fit (solid lines) monitored at 690 nm wavelength for Sb‐rich and Se‐rich Sb_2_Se_3_ films.

To figure out the effect of deep‐level defect on the carrier transport dynamics, we then carry out TAS characterizations. The time window of time‐resolved transient spectra is of 0–2000 ps. A 360 nm pulse laser is selected as source to illuminate the Sb_2_Se_3_ films deposited on soda lime glass. According to the TAS mapping (**Figure** **5**a,b), we find that there are only one absorption peaks near 690 nm wavelength detected in both films, which belongs to the characteristic absorption peak of Sb_2_Se_3_. Thereout, we obtain the decay kinetics of two films tracked at 690 nm wavelength (Figure 5c). Additionally, due to the absence of electron donor and acceptor layer, the decay kinetics reflects the whole relaxation of exciton in Sb_2_Se_3_ to ground state through recombination.^[^ ^22^ ^]^ Finally, the kinetics is fitted by biexponential decay model, and the results are listed in Table S3 (Supporting Information). The carrier lifetime for Sb‐rich and Se‐rich Sb_2_Se_3_ are 7.66 and 16.15 ns, respectively. The increased carrier lifetime of Se‐rich Sb_2_Se_3_ exactly suggests the less defect induced SRH recombination compared with Sb‐rich. Hence, TAS study consolidates the deep‐level defect analysis and interprets the enhanced photovoltaic performance and photoelectric response (Figure 3b) in Se‐rich device.

To further study effects of Sb_2_Se_3_ composition on deep‐level defects, we also prepare Sb_2_Se_3_ films with Se/Sb atomic ratio of 1.50 (stochiometric) and 1.60 (high Se‐rich) by delicately tuning the addition of Se or Sb during thermal evaporation (Table S4, Supporting Information). Then we conduct DLTS and TAS measurements for stoichiometric and high Se‐rich Sb_2_Se_3_ (Table S5, Figures S5 and S6, Supporting Information). The DLTS results demonstrate that stoichiometric and high Se‐rich Sb_2_Se_3_ possess similar trap states with Se‐rich (Se/Sb ≈1.56). They all exhibit two hole traps H2 and H3 corresponding to V_Sb_ and Se_Sb_ defect. Additionally, the trap density of stoichiometric Sb_2_Se_3_ is much approximate to Se‐rich condition, correspondingly, whose carrier lifetime (15.58 ns) gained from TAS is also close to that of Se‐rich (16.15 ns). It implies that the variation of element composition within a certain range, i.e., close to the stochiometric and Se‐rich Sb_2_Se_3_, may not lead to essential changes in the deep‐level defect types. However, the trap density of V_Sb_ and Se_Sb_ are increased with the higher Se/Sb atomic ratio, which induce reduced carrier lifetime.

To establish the correlation between SRH recombination, *V*_OC_ loss and defect properties, we examine the dark *J*–*V* curves of optimal Sb‐rich and Se‐rich devices (Figure S7, Supporting Information). In comparison, the Se‐rich Sb_2_Se_3_ device delivers lower ideality factor (*A* = 1.47) and reverse saturate current (*J*_0_ = 2.15 × 10^−4^ mA cm^−2^) than that of Sb‐rich (*A* = 1.62, *J*_0_ = 7.36 × 10^−3^ mA cm^−2^). In principle, *A* and *J*_0_ are strongly related to the rectification of diode. The ideality factor value falls into 1 to 2, suggesting the hybrid recombination mechanism from interface and bulk simultaneously.^[^ ^13^ ^]^ Besides, the reduced *A* value for Se‐rich device suggest the more efficient suppression of trap‐assisted SRH recombination in bulk.^[^ ^23^ ^]^

In addition, since the *V*_OC_ is inversely associated with *J*_0_ in line with Equation (1), the reduced *J*_0_ in Se‐rich device also contribute to enhanced photovoltaics performance.

(1) $$V_{\mathrm{OC}}=\frac{Ak_{B}T}{q}\;\ln\left(\frac{J_{\mathrm{SC}}}{J_{0}}+1\right)$$

where *k*_B_, *T*, and *q* are Boltzmann constant, temperature, and elementary charge, respectively. In practice, *J*_0_ consists of two parts, one is the leakage current caused by interface recombination (*J*IF 0), and the other is leakage current caused by SRH recombination in space charge region (SCR, *J*SCR 0) according to Equation (2).^[^ ^24^ ^]^

(2) $$J_{0}=J_{0}^{\mathrm{IF}}\;+J_{0}^{\mathrm{SCR}}$$

(3) $$J_{0}^{\mathrm{SCR}}=\frac{\pi k_{B}T\sqrt{N_{C}N_{V}}}{2\tau F_{m}}\;\exp\left(-\frac{E_{g}}{2k_{B}T}\right)$$

Furthermore, the *J*SCR 0 is inversely proportional to carrier lifetime (Equation (3), the *N*_C/V_, *F*_m_, and *E*_g_ are state density in conduction (valence) band, the electric field at the position of maximum recombination and band gap.).^[^ ^13^ ^]^ Accordingly, we can draw a conclusion that *V*_OC_ is positively related to carrier lifetime. It suggests the decrescent carrier lifetime stemming from the defect‐induced SRH recombination is the predominant cause for *V*_OC_ loss. Notably, Se‐rich Sb_2_Se_3_ possesses relatively lower defect concentration and prolonged carrier lifetime when compared with Sb‐rich once, indicating that the Se‐rich Sb_2_Se_3_ is more promising for obtaining high efficiency device in terms of increasing the carrier lifetime and resultant *V*_OC_.^[^ ^13^ ^]^

## 3 Conclusion

In summary, we reveal the stoichiometry depended defect properties in Sb_2_Se_3_ films. We confirmed the purity of the as‐synthesized Sb_2_Se_3_ films in terms of crystal structure, compositional elements as well as the chemical bond, which provides solid ground for defect analysis. We experimentally disclose the theoretically predicted amphoteric Sb_Se_ defect in Sb‐Sb_2_Se_3_, enriching the fundamental understanding in the defect study. We also provide new perspective in different challenges in the Sb‐rich and Se‐rich Sb_2_Se_3_ based solar cells. In Se‐rich Sb_2_Se_3_ solar cell, it has relatively low defect concentration and shallower energy level, showing promise in the efficiency improvement. However, although the Sb‐rich Sb_2_Se_3_ sample possesses two kinds of defect, they are actually coming from one kind of element substitution. Practically, it simplifies the defect engineering in materials synthesis.

## 4 Experimental Section

### Preparation of Sb_2_Se_3_ Films

Sb_2_Se_3_ films were deposited on substrate using thermal evaporation deposition under a pressure of 5 × 10^−4^ Pa. Sb_2_Se_3_ (99.99%, zhongnuoxincai) powder was used as evaporation source, and an amount of Sb or Se (99.999%, Sinopharm) powder was used as co‐evaporation source to tailor the composition of Sb_2_Se_3_ film. The Sb_2_Se_3_ vapor was deposited on substrate (315 °C preheated) with an evaporation rate of 4–6 nm s^−1^. The final thickness of films was controlled around 300 nm. Finally, the as‐deposited films were post‐annealed at 380 °C for 8 min on a preheated hot plate in a N_2_‐filled glove box.

### Fabrication of Sb_2_Se_3_ Solar Cells

The FTO glass (TEC‐A7) was cleaned by DI water, isopropanol, acetone, and ethanol firstly, and then was cleaned for 15 min by UV ozone. Next, a 60‐nm CdS ETL was deposited on FTO by CBD method.^[^ ^25^ ^]^ Subsequently, the Sb_2_Se_3_ film was deposited on FTO/CdS substrate by thermal evaporation method as described above. Afterwards, 90‐nm thick Spiro‐OMeTAD (doped with 520 mg mL^−1^ Li‐TFSI acetonitrile solution) layer was selected as HTL.^[^ ^25^ ^]^ Finally, the 60‐nm Au back electrode was evaporated on the HTL under a pressure of 5 × 10^−4^ Pa. The active area was defined as 0.04 cm^−2^ by a metallic mask.

### Characterization of Films and Devices

The SEM images of Sb_2_Se_3_ thin films were characterized by FE‐SEM (Hitachi SU8220) equipped with an EDS (Bruker) module. The AFM topographics for Sb_2_Se_3_ films were characterized by Bruker Dimension Icon. The crystal structure was measured by XRD (Bruker Advance D8 diffractometer) with Cu K*α* radiation (*λ* = 1.5406 Å). Raman spectroscopy was carried out using a Renishaw Raman spectrometer with 532 nm laser excitation. XPS spectra of the films were conducted on a Thermo Scientific K‐Alpha^+^ instrument equipped with a monochromatic Al K*α* X‐ray source. UV−vis spectrophotometer (SOLID3700) was used to measure the light absorption properties of the Sb_2_Se_3_ films. UPS spectra was conducted via a PHI5000 VersaProbe III (Scanning ESCA Microprobe) SCA (Spherical Analyzer), and a He (I) discharge lamp (21.2 eV) was selected as the UV light source. The work function was calculated by subtracting the secondary electron cutoffs from the excitation light energy (21.2 eV). The Hall effect measurement was exerted by Ecopia Hall Effect Tester (HMS‐7000) at 300 K. TAS of Sb_2_Se_3_ films were measured by a commercial Helios setup from Ultrafast System. Additionally, the pump and probe laser pulses were generated by frequency doubling the fundamental output (Coherent Vitesse, 80 MHz, Ti‐sapphire laser) and through white light generated with a sapphire plate, respectively. The decay kinetics were fitted by biexponential model *y* = Σ*A*_i_exp(−*x*/*t*_i_), and carrier lifetime (*τ*) was obtained by *τ* = Σ*A*_i_ *t*_i_^2^/Σ*A*_i_ *t*_i_ (*i* = 2). A Keithley 2400 apparatus along with solar‐simulated AM 1.5 G sunlight (a standard xenon‐lamp‐based solar simulator (Oriel Sol 3A)) was used to examine the *J–V* characteristics. The solar simulator was calibrated before test by a standard monocrystalline silicon (Oriel P/N 91150 V, with a KG‐5 visible color filter), calibrated by NREL. The EQE values of the devices were measured using an illumination system with a single source (halogen lamp) in connection with a monochromator (Model SPIEQ200). EIS was performed at a bias of 0.2 V in dark with the frequency ranging from 1 MHz to 1 Hz based on Zahner Mess System PP211 electrochemical workstation. The DLTS measurement was performed via a Phystech FT‐1230 HERA‐DLTS system equipped with a 10‐mW red laser (635 nm wavelength). The modified Boonton 7200 capacitance meter was used to measure dynamic capacitance. A complete device with area of 0.01 cm^−2^ was selected to test. The temperature scan range was from 120 to 420 K at 2 K heating intervals. The dual pulse mode was set as electrical (pulse voltage) and optical (laser excitation), and they were exerted and removed simultaneously. In specific, the reverse bias, pulse voltage, pulse width (electric & optical), and period width were −0.4 V, 0.2–0.6 V, 10 ms, and 100 ms, respectively.

## Conflict of Interest

The authors declare no conflict of interest.

## Supporting information

Supporting Information

## Data Availability

The data that support the findings of this study are available from the corresponding author upon reasonable request.
