# Supplementary material for: Distinctive Deep‐Level Defects in Non‐Stoichiometric Sb2Se3 Photovoltaic Materials
Source: Adv Sci (Weinh). 2022 Jan 25;9(9):2105268. doi: 10.1002/advs.202105268 (PMC8948662; doi:10.1002/advs.202105268)
Supplement: Supplementary file 1 — Supporting Information [file ADVS-9-2105268-s001.pdf]

ADVANCED SCIENCE
Open Access

Supporting Information

for *Adv. Sci.*, DOI 10.1002/advs.202105268

Distinctive Deep-Level Defects in Non-Stoichiometric Sb_2Se_3 Photovoltaic Materials

*Weitao Lian, Rui Cao, Gang Li, Huiling Cai, Zhiyuan Cai, Rongfeng Tang, Changfei Zhu, Shangfeng Yang and Tao Chen**

ADVANCED SCIENCE
Open Access

Supporting Information

for *Adv. Sci.*, DOI: 10.1002/advs.202105268

Distinctive Deep-Level Defects in Non-Stoichiometric Sb_2Se_3 Photovoltaic Materials

*Weitao Lian, Rui Cao, Gang Li, Huiling Cai, Zhiyuan Cai, Rongfeng Tang, Changfei Zhu, Shangfeng Yang and Tao Chen**

Supporting Information

Distinctive Deep-Level Defects in Non-Stoichiometric Sb_2Se_3 Photovoltaic Materials

*Weitao Lian, Rui Cao, Gang Li, Huiling Cai, Zhiyuan Cai, Rongfeng Tang, Changfei Zhu, Shangfeng Yang and Tao Chen**

W. T. Lian, R. Cao, G. Li, H. L. Cai, Z. Y. Cai, R. F. Tang, C. F. Zhu, S. F. Yang, T. Chen

Hefei National Laboratory for Physical Sciences at Microscale, CAS Key Laboratory of Materials for Energy Conversion, Department of Materials Science and Engineering, School of Chemistry and Materials Science, University of Science and Technology of China, Hefei, Anhui 230026, P. R. China

Institute of Energy, Hefei Comprehensive National Science Center, Hefei, China

E-mail: tchenmse@ustc.edu.cn

Key words: antimony selenide, deep-level defect, SRH recombination, carrier lifetime, DLTS

Table S1. The statistical composition information by EDX spectra for each 10 samples of Sb-rich and Se-rich Sb_2Se_3 films.

**Sb_2Se_3 films**	**Sb (at%)**	**Se (at%)**	**Se/Sb**
Sb-rich	42.28±0.75	57.72±0.75	1.37±0.04
Se-rich	39.09±0.76	60.91±0.76	1.56±0.05

Table S2. Hall effect measurements for Sb-rich and Se-rich Sb_2Se_3 films at 300 K.

**Sb_2Se_3 films**	**Mobility (cm^2 V^{-1} S^{-1})**	**Carrier density (cm^{-3})**	**Hall coefficient (cm^3 C^{-1})**	**Resistivity (Ω cm)**
Sb-rich	3.26	1.07×10^{16}	-4.64×10^{5}	27.3
Se-rich	3.09	1.05×10^{16}	-3.79×10^{2}	34.7

Table S3. Biexponential fitting results of TAS monitored at 690 nm wavelength.

**Sb_2Se_3 films**	**A_1**	**t_1 (ns)**	**A_2**	**t_2 (ns)**	**τ (ns)**
Sb-rich	0.68	0.04	0.32	7.74	7.66
Se-rich	0.55	0.13	0.45	16.31	16.15

Table S4. The statistical composition information from EDX spectra for each 10 samples of stoichiometric and high Se-rich Sb_2Se_3 films.

**Sb_2Se_3 films**	**Sb (at%)**	**Se (at%)**	**Se/Sb**
Stoichiometric	40.03±0.24	59.97±0.24	1.50±0.02
high Se-rich	38.52±0.55	61.48±0.55	1.60±0.04

Table S5. The E_T, σ and N_T extracted from DLTS measurement for stochiometric (Se/Sb ~1.50) and high Se-rich Sb_2Se_3 (Se/Sb ~1.60).

Sb_2Se_3	Trap level	E_T (eV)	σ (cm^2)	N_T (cm^{-3})
Se/Sb ~1.50	H2	E_V+0.52±0.06	(0.08~3.62)×10^{-15}	(0.37~1.81) ×10^{13}
	H3	E_V+0.67±0.08	(0.18~4.89) ×10^{-16}	(1.32~3.88) ×10^{13}
Se/Sb ~1.60	H2	E_V+0.50±0.03	(1.61~9.13) ×10^{-17}	(0.55~4.78) ×10^{13}
	H3	E_V+0.67±0.05	(0.02~3.25) ×10^{-15}	(2.36~6.75) ×10^{13}

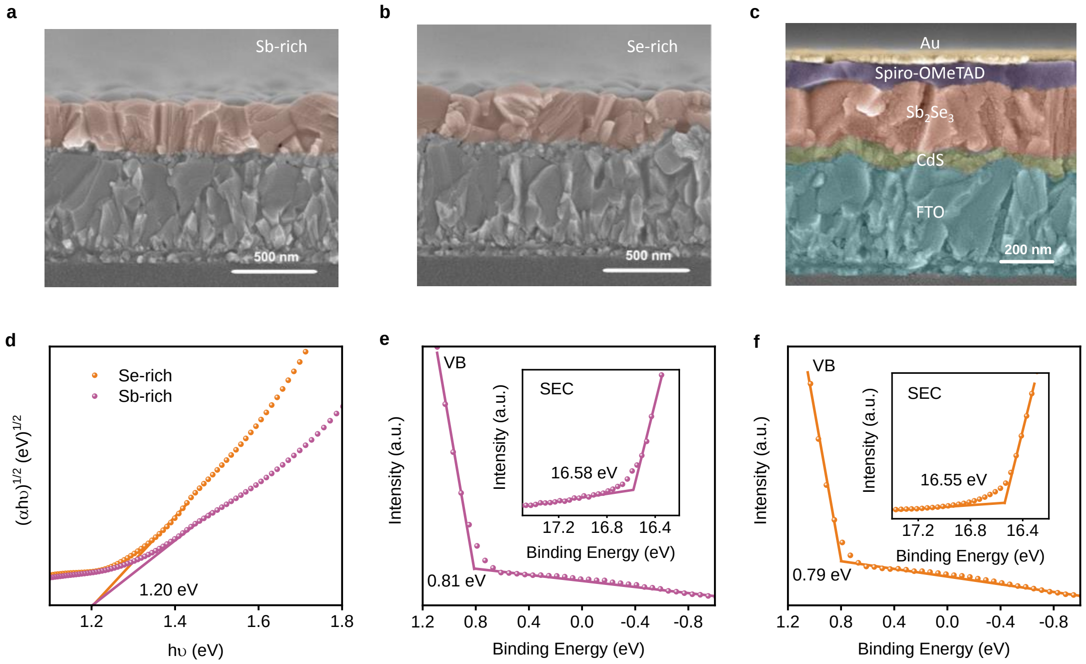

Figure S1. a-b) Cross-sectional SEM images of Sb-rich and Se-rich Sb_2Se_3 films. c) Cross-sectional SEM images of planar Sb_2Se_3 solar cells. d) UV-vis spectroscopy of Sb and Se-rich Sb_2Se_3 films. e-f) Valence band (VB) position and secondary cutoff (SEC) from UPS spectra of Sb-rich and Se-rich Sb_2Se_3 films.

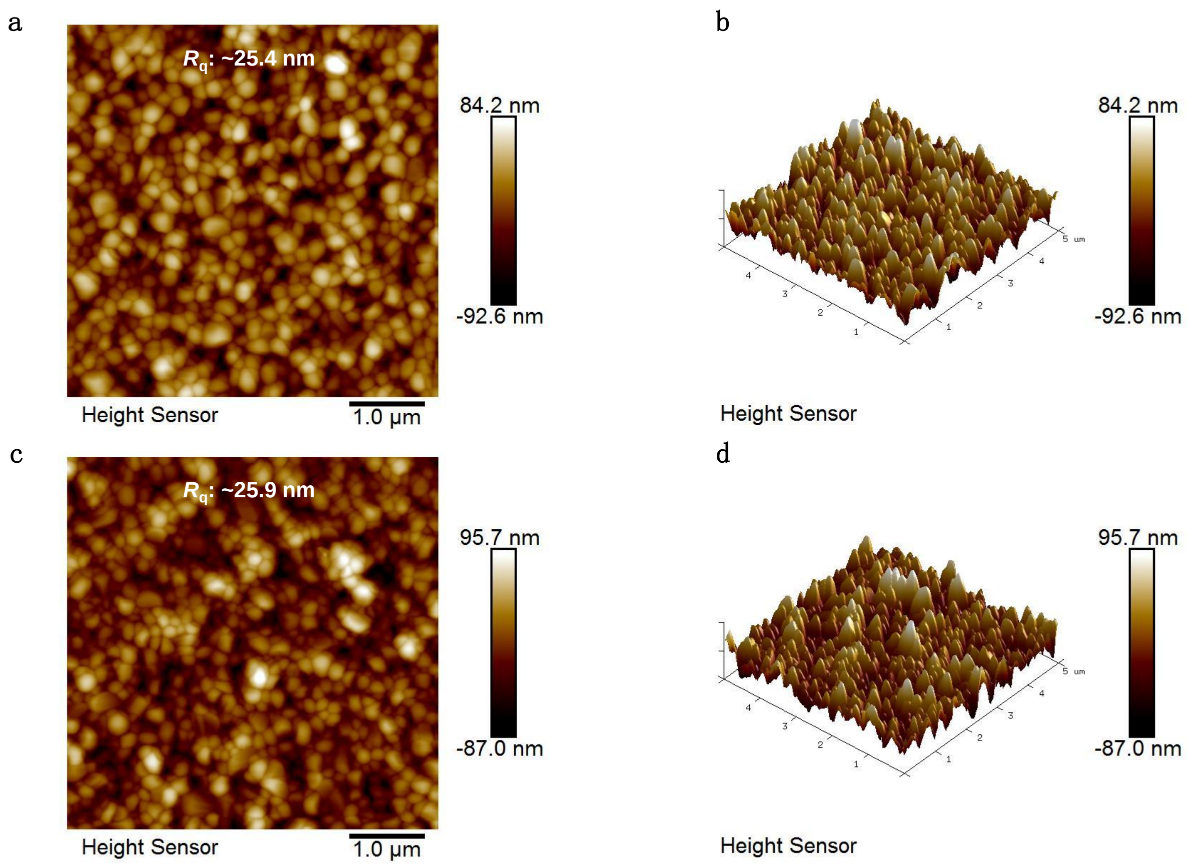

Figure S2. a-b) 2D and 3D AFM images for Sb-rich Sb_2Se_3 films. c-d) 2D and 3D AFM images for Se-rich Sb_2Se_3 films.

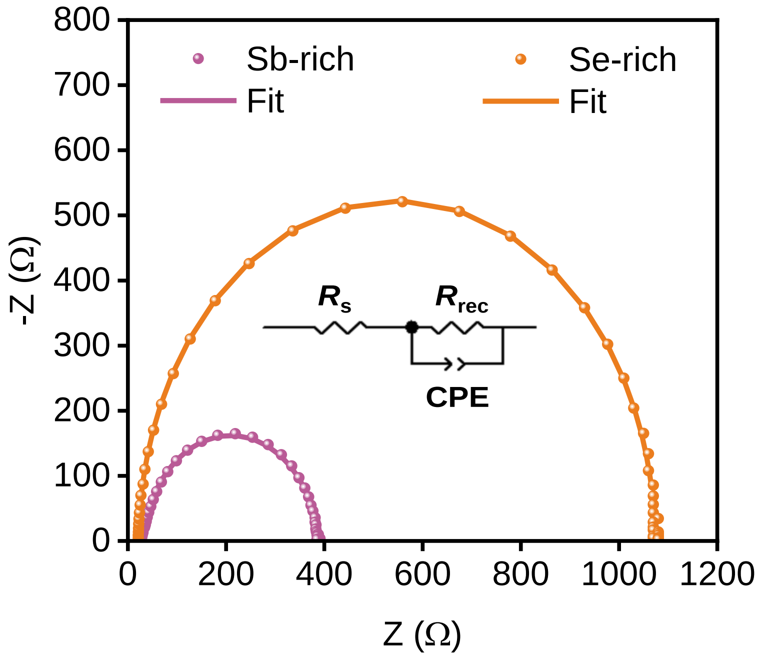

Figure S3. EIS measurement for Sb-rich and Se-rich Sb_2Se_3 device.

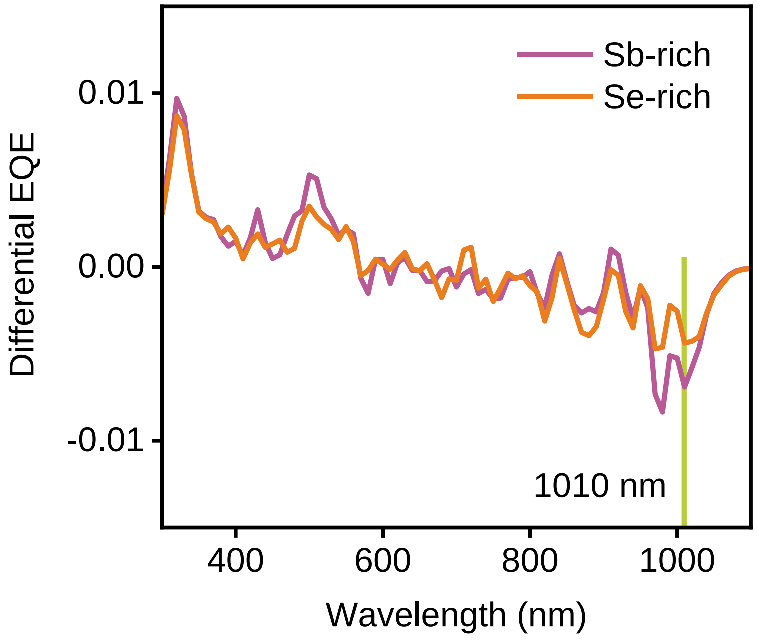

Figure S4. Differential EQE spectra of Sb-rich and Se-rich Sb_2Se_3 films.

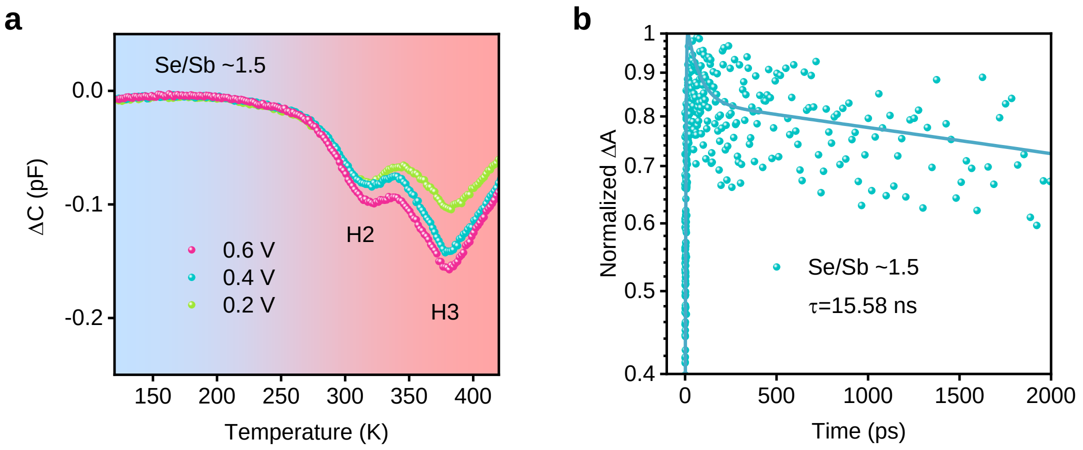

Figure S5. a-b) DLTS signals and TAS measurement for stochiometric Sb_2Se_3 (Se/Sb ~1.50).

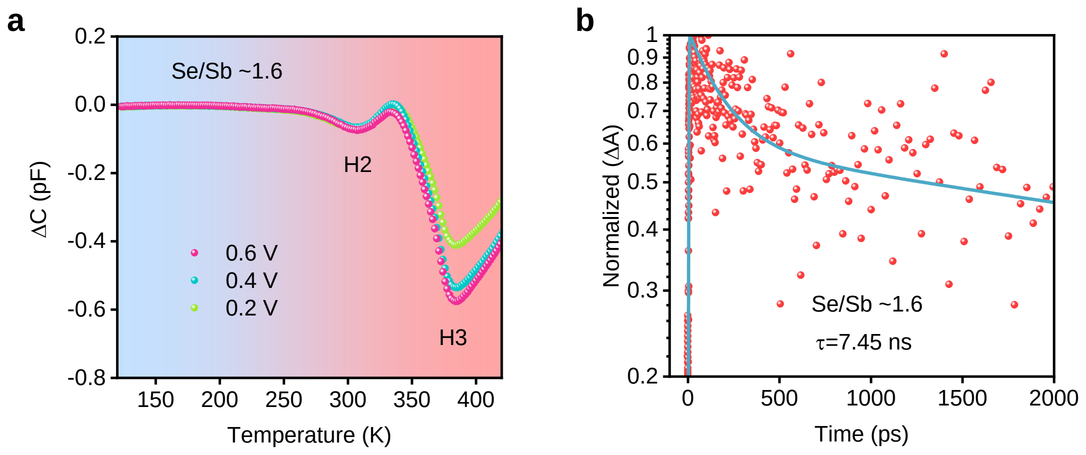

Figure S6. a-b) DLTS signals and TAS measurement for high Se-rich Sb_2Se_3 (Se/Sb ~1.60).

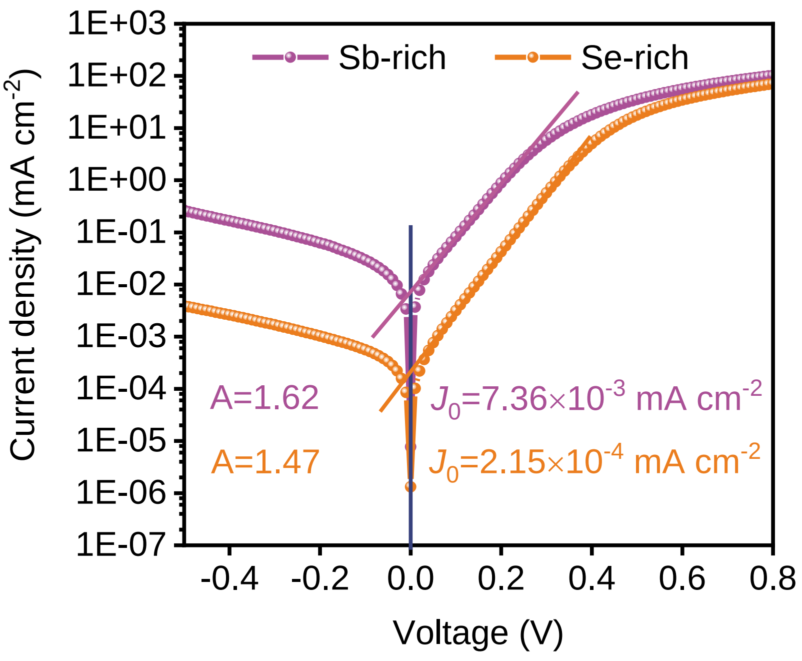

Figure S7. Dark *J*-*V* curves of Sb-rich and Se-rich Sb_2Se_3 solar cells.

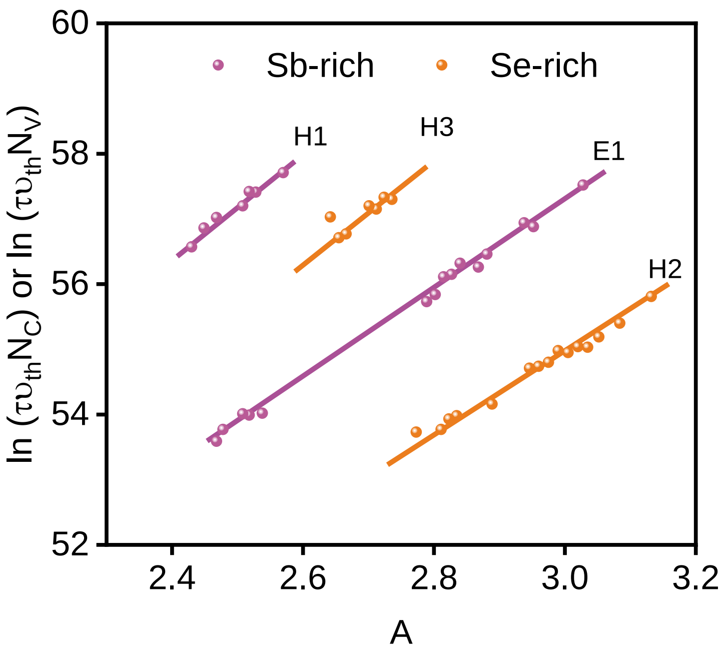

Figure S8. Linear Arrhenius plots obtained from DLTS signals for Sb-rich and Se-rich devices.

The Arrhenius plots are calculated by Equation (1) and (2) obtained from DLTS signals, and the active energy (E_a, E_C-E_T or E_T-E_V) and capture cross section of traps can be extracted.

$$\ln(\tau_e \upsilon_{th,n} N_C) = \frac{E_C - E_T}{k_B T} - \ln(X_n \sigma_n)\,,\#(1)$$

$$\ln(\tau_e \upsilon_{th,p} N_V) = \frac{E_T - E_V}{k_B T} - \ln(X_p \sigma_p)\,.\#(2)$$

where τ_e, N_C and N_V are emission time constant, conduction band state density and valence band state density, respectively. $\upsilon_{th,n/p}$, $X_{n/p}$ and $\sigma_{n/p}$ represent thermal velocity, entropy factor and capture cross section for electron and hole, respectively. In addition, the trap density (N_T) could be obtained by Equation (3),

$$N_T = 2N_S \frac{\Delta C}{C_R}\,.\#(3)$$

where N_S is the shallow donor concentration, C_R is the capacitance under reverse bias, while ΔC represents the amplitude of transient capacitance.
